# Effectively teaching cultural competence in a pre-professional healthcare curriculum

**DOI:** 10.1186/s12909-024-05507-x

**Published:** 2024-05-21

**Authors:** Karen R. Bottenfield, Maura A. Kelley, Shelby Ferebee, Andrew N. Best, David Flynn, Theresa A. Davies

**Affiliations:** 1 Graduate Medical Sciences, Boston University Chobanian & Avedisian School of Medicine, 72 East Concord Street, L317, R-1017, Boston, MA 02118 USA; 2 Department of Medical Sciences & Education, Boston University Chobanian & Avedisian School of Medicine, 72 East Concord Street, Boston, MA 02118 USA; 3 University of Maryland School of Dentistry, 650 W Baltimore Street, Baltimore, MD 21201 USA

**Keywords:** Teaching, Culture, Competence, Healthcare, Curriculum, Predental, Premedical, Communication, Learning

## Abstract

**Background:**

There has been research documenting the rising numbers of racial and ethnic minority groups in the United States. With this rise, there is increasing concern over the health disparities that often affect these populations. Attention has turned to how clinicians can improve health outcomes and how the need exists to educate healthcare professionals on the practice of cultural competence. Here we present one successful approach for teaching cultural competence in the healthcare curriculum with the development of an educational session on cultural competence consisting of case-based, role-play exercises, class group discussions, online discussion boards, and a lecture PowerPoint presentation.

**Methods:**

Cultural competence sessions were delivered in a pre-dental master’s program to 178 students between 2017 and 2020. From 2017 to 2019, the sessions were implemented as in-person, case-based, role-play exercises. In 2020, due to in-person limitations caused by the COVID-19 pandemic, students were asked to read the role-play cases and provide a reflection response using the online Blackboard Learn discussion board platform. Evaluation of each session was performed using post-session survey data.

**Results:**

Self-reported results from 2017 to 2020 revealed that the role-play exercises improved participant’s understanding of components of cultural competence such as communication in patient encounters (95%), building rapport with patients (94%), improving patient interview skills (95%), and recognition of students own cultural biases when working with patients (93%).

**Conclusions:**

Students were able to expand their cultural awareness and humility after completion of both iterations of the course session from 2017 to 2019 and 2020. This session can be an effective method for training healthcare professionals on cultural competence.

**Supplementary Information:**

The online version contains supplementary material available at 10.1186/s12909-024-05507-x.

## Background

It is projected that by the year 2050, racial and ethnic minority groups will make up over 50% of the United States population [1]. With a more multicultural society, growing concern has emerged over how to address the health disparities that effect these populations and the ways in which healthcare professionals can increase positive health outcomes. Continuing evidence suggests that many patients from racial and ethnic minority groups are not satisfied with the current state of healthcare which has been attributed to implicit bias on the part of physicians and current challenges faced by practitioners who feel underprepared to address these issues due to differences in language, financial status, and healthcare practice [2–4].

To contend with health disparities and the challenges faced by practitioners working with a more diverse population, healthcare educators have begun to emphasize the importance of educating healthcare workforce on the practice of cultural competence and developing a skilled-based set of behaviors, attitudes and policies that effectively provides care in the wake of cross-cultural situations and differences [4–6]. There are several curricular mandates from both medical and dental accreditation bodies to address this issue [7–9], and large amounts of resources, ideas, and frameworks that exist for implementing and training future and current healthcare providers on the inadequacies of the healthcare system and cultural competence [10–12]. These current institutional guidelines for accreditation and the numerous amounts of resources for training cultural competence, continue to evolve with work documenting the need for blended curriculum that is continuous throughout student education, starting early as we have done here with pre-dental students, including in-person didactic or online sessions, a service learning component, community engagement and a reflective component [4, 5, 13, 14].

This study investigates teaching cultural competence in a healthcare curriculum. We hypothesized that early educational exposure to cultural competence through role playing case studies, can serve as an effective mechanism for training early pre-doctoral students the practice of cultural competence. Utilizing student self-reported survey data conducted in a predental master’s curriculum, in which two iterations of role-playing case studies were used to teach components of cultural competence, this study aims to evaluate and support research that suggests role-playing case studies as effective means for educating future clinical professionals on the practice of cultural competence.

## Methods

### Ethics

This study was determined to be exempt by the Institutional Review Board of Boston University Medical Campus, Protocol # H-37,232. Informed consent was received from all subjects.

### Data collection

The role-playing, case-based simulated patient encounter exercises were developed and administered at Boston University Chobanian & Avedisian School of Medicine to predental students in the Master of Science in Oral Health Sciences Program (see Table 1). From 2017 to 2020, we administered patient encounter cases [see Additional File 1] to students (*n* = 178) in the program as a portion of a case-based, role-playing exercise to teach the importance of cultural competence and cultural awareness during patient encounters. During years 2017–2019, real actors portrayed the patient and physician. In 2020, the session was conducted online via a discussion board through a Blackboard Course Site. The original case was published as part of a master’s students thesis work in 2021 [15].

**Table 1:** Summary of 2020 discussion board themes and reflection responses following cultural competency sessions

Theme	Number of Responses			Percent of Responses (%)	Representative Reflection Responses
	1 A & 1B	2 A & 2B	Total		
Understanding a Holistic Approach to Care	71	57	128	29.0%	“Being a clinician means more than treating the illness, it means treating the whole person.”
					“…the provider should consider the overall needs of the patient in order to treat the patient to the best of their ability.”
					“It is essential for the physician to learn about the patient and develop a good understanding of their health, lifestyle, and well-being.”
					“Shows that the clinician has chosen a more holistic approach and was trying to provide the patient with the appropriate resources that could help her quality of life and overall health.”
Understanding the Importance of taking a Patient History	69	38	107	24.3	“The clinician in 2A missed a lot of important information about the patient just because they didn’t take the time to ask a few simple questions.”
					“In scenario 2A, again the clinician addresses the immediate health concern without establishing a comprehensive patient health history.”
					“made me realize how important it is to ask the simple questions, because they can tell you a lot. From the encounter, a care provider would never have known the patient was homeless or HIV positive, which is extremely important to note in the patient’s history…”
					“In scenario 1B, a more detailed assessment was given with more detailed patient information regarding the patient’s personal life, sexual history, lack of access to healthcare, lack of employment, or residence of living.”
Recognition of Socioeconomic Factors During a Patient Encounter	30	30	30	6.8	“Underserved populations and patients often don’t have the resources or often the knowledge on how to get out of their current situation.”
					“The patient in encounter 1, is displaying a large array of problems in her life aside from the UTI; she lost her child, she is homeless, and she is financially unstable.”
					“I think focusing on the patient’s socioeconomic status, and their level of engagement is key when diagnosing patients.”
Understanding the Importance of Patient Clinician Relationship	61	62	123	27.9	“In scenario 1B, the clinician shows empathy toward the patient…”
					“I think that scenario 1 emphasizes that being a clinician goes way beyond just knowing medical facts and that a very large part of healthcare is also relating to people one-on-one.”
					“Scenario 1B the physician made sure the patient was comfortable. Overall, scenario 1B is a more ideal situation because the conversation is more patient focused and has a good balance of the patient’s needs and the providers thoughts.”
Improvement of Health Outcomes	27	26	53	12.0	“Really taking the time to explain what the patient’s medical condition [is] can impact how they respond to follow-up treatment.”
					“The patient in 1B receives better care.”
					“I think in scenario 2, the doctor does a better job at providing care for their patient which leads to better outcomes and compliance to attend follow-up appointments.”
Total	258	213	441	100	

Themes coded using NVIVO following discussion board Scenario 1 A & 1B reflections and comments for 40 students (441 reflections/responses)

### Description of patient encounter cases 1 and 2

Patient Encounter Case 1 [see Additional file 1] is composed of two subsections, scenario 1 A and scenario 1B, and is centered around a patient/physician interaction in which a patient who is pregnant presents with pain upon urination. The physician in 1 A is short and terse with the patient, immediately looking at a urine sample, prescribing medication for a urinary tract infection, and telling the patient to return for a follow-up in 2 weeks. In scenario 1B, a similar situation ensues; however, in this scenario the physician takes more time with the patient providing similar care as the physician in 1 A, but asking for more information about the patients personal and medical history. At the conclusion of the scenario, the patient is offered resources for an obstetrician and a dentist based on the information that is provided about the patient’s background. The patient is then sent on their way and asked to follow-up in 2 weeks. The patient does not return.

Patient Encounter Case 2 [see Additional file 1] follows a similar format to the Patient Encounter Case 1. In scenario 2 A, the same patient from Case 1 returns with tooth pain after giving birth. The physician in 2 A, like 1 A, is short with the patient and quickly refers the patient to a dentist. In 2B, the physician again takes more time with the patient to receive background information on the patient, make a connection, and provides an antibiotic and dental referral.

Each Patient Encounter Case explored topics such as the importance of building a trusting physician/patient relationship, the importance of asking a patient for patient history, making a connection, and the importance of a physician taking all facets of a patient’s circumstances into consideration [15].

### Session outline

The sessions conducted between 2017 and 2019 were composed of three parts: (1) enactment of an abridged patient encounter facilitated by session administrators, (2) group discussion and reflection during which time students were asked to critically reflect and discuss the theme and key take-aways from the role play exercise, and (3) a PowerPoint presentation emphasizing take-away points from the role-play exercise. At the conclusion of the cultural competence training sessions, students participated in a post-session Qualtrics generated survey administered electronically to assess each student’s feelings about the session [see Additional file 3].

### Role-play enactment

Facilitators dressed-up in clothing to mimic both the physician and patient for all case scenarios in Patient Encounter Case 1 and Case 2. At the conclusion of the role play portion of each of the cases, the facilitators paused to lead students in a real-time class group discussion. After Case 1, students were asked questions such as: *What did you think*? *Were the patient’s needs met? Did you expect the patient to return?* Following Case 2, similar questions were asked by the facilitators, including: *What did you think*? *Were the patient’s needs met? Did you expect the patient to accept help?*

At the conclusion of this portion of the session, the facilitators led a larger general discussion about both cases and how they related to one another. Finally, the course session concluded with a PowerPoint presentation that reinforced the take-home points from the session [see Additional file 2] [15].

### Change in session modality due to COVID-19 pandemic

In Fall 2020, due to the COVID-19 pandemic, the course modality moved to an online platform and consisted of three parts on a Blackboard Discussion Board (Blackboard, Inc.). Students were required to: (1) read each of the Patient Encounter Cases and add a brief reflection comparing the scenarios, (2) then comment on at least two peer’s posts in the discussion forum and (3) attend class to hear a PowerPoint presentation by a course session facilitator on the key take-aways from each scenario [15].

### Student surveys

At the conclusion of the cultural competence training sessions, students participated in a post-session Qualtrics (https://www.qualtrics.com) generated survey administered electronically to assess each student’s feelings about the sessions [see Additional file 3]. The format of the survey included 5 questions with the following Likert scale response options: strongly agree, agree, disagree, strongly disagree. These post-session surveys were not required but rather optional [15].

## Results

A total of 178 students completed the cultural competence sessions between 2017 and 2020. Of these participants, 112 voluntarily completed a post-session survey on the effectiveness of the course in teaching cultural competence and cultural awareness during patient encounters. Between 2017 and 2019, 99 students completed post-session surveys following sessions with role play exercises. In 2020, 13 students completed post-session surveys following discussion board sessions.

### Role-play exercises enhanced cultural competence

**Fig. 1:**
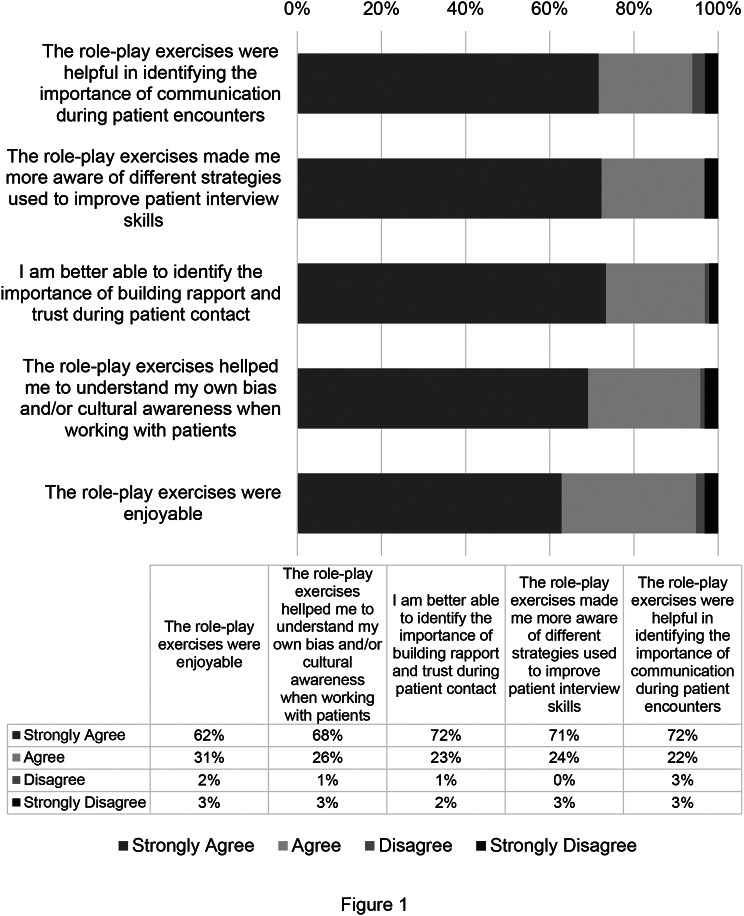
Cultural Competence Session Survey Data from the Year 2017–2019. Survey data from students at Boston University’s Oral Health Sciences Program for the years 2017–2019. Data is presented as percent of respondents (*n* = 99)

In responding to post-session survey questions following cultural competence sessions that included role-play exercises (2017–2019), 71% of students surveyed strongly agreed and 24% agreed that the role-play exercises helped them to identify the importance of communication in patient encounters. In asking participants if the role-play exercises made them more aware of different strategies to improve their patient interview skills, 72% strongly agreed and 23% agreed. Also, 68% of the students strongly agreed and 26% agreed that the exercises helped them to better identify the importance of building rapport and trust during patient encounters. When asked if the exercises helped the students to better understand their own bias and/or cultural awareness when working with patients, the results of the survey showed that 62% of students strongly agreed and 31% agreed with this statement. In addition, most students found the role-play exercises to be enjoyable (72% strongly agreed and 22% agreed). See results shown in Fig. 1.

### Discussion boards and reflections enhanced cultural competence

**Fig. 2:**
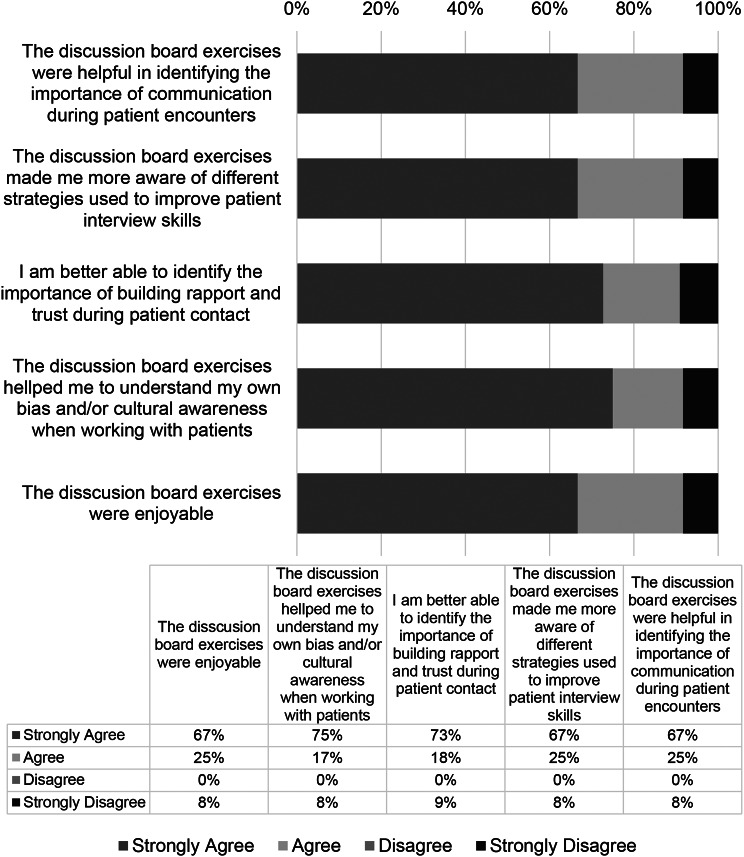
Cultural competence session survey data from the Year 2020. Survey data from students at Boston University’s Oral Health Sciences Program for the year 2020. Data is presented as percent of respondents (*n* = 13)

Cultural competence sessions held during 2020 did not include role-play exercises due to the Covid-19 pandemic. Instead, students participated in discussion boards and reflections on Blackboard. In response to the post-session survey question asking if the discussion board exercises were helpful in identifying the importance of communication during patient encounters, 67% of students strongly agreed and 25% agreed with this statement. Also, 75% of students strongly agreed and 17% agreed that the discussion board exercises helped them identify the importance of building rapport and trust during patient contact. When asked if the exercises helped the students to better understand their own bias and/or cultural awareness when working with patients, the results of the survey showed that 67% of students strongly agreed and 25% agreed with this statement. In addition, most students found the discussion board exercises to be enjoyable (67% strongly agreed and 22% agreed). See results shown in Fig. 2.

Student responses to the reflection portion of the online cultural competency sessions were recorded and categorized. Five themes were selected and 441 reflection responses were coded using NVivo (Version 12). The results showed that 29% of reflections demonstrated student’s ability to understand a holistic approach to clinical care, 24.3% understood the importance of collecting a patient history, 6.8% recognized the socioeconomic factors during a patient encounter, 27.9% reflected on the importance of the patient clinical relationship, and 12% on the effects on improving health outcomes (Table 1). Representative student responses to these themes are shown in Table 1.

## Discussion

There exists a need to develop novel and effective means for teaching and training the next generation of healthcare professionals the practice of cultural competence. Thus, two iterations of a course session using case-based patient centered encounters were developed to teach these skills to pre-professional dentals students. Overall, the results of this study demonstrated that participation in the course, subsequent group discussion sessions, and take-away PowerPoint sessions significantly improved the participant’s understanding of the importance of communication skills and understanding of socioeconomic, environmental, and cultural disparities that can affect a patient’s health outcome.

According to results from the course session implemented in-person from 2017 to 2019, the role-playing exercise significantly improved participants understanding of important components that can be used to improve health outcomes that may be affected due to health disparities. Students were strongly able to identify the importance of communication in patient encounters, to understand strategies such as communication and compassionate care in patient encounters, identify the importance of building a patient-physician relationship with patients, and were able to recognize their own cultural biases. Similarly, in 2020, even with a change in course modality to on-line learning due to COVID-19, students were able to understand the same key take-aways from the course session as demonstrated by reflections using the discussion board regarding the need for a holistic approach to care, importance of the patient clinician relationship, and importance of taking a patient history. Despite promising implications of both iterations of the session, students completing the session online did not find the same success in “understanding my own bias/and or cultural awareness when working with patients.” This decrease may be attributed to change in course modality and the strengths of the role-play enactment of the patient encounter. It is important to recognize that additional learning components, including video recordings of the role-play enactment, may be necessary if the discussion board is used as the primary learning method in the future.

In contrast to previous studies that attempted to determine the effectiveness of cultural competence training methods, this session had many unique characteristics. The simulated role-playing exercise enabled student participants to see first-hand an interactive patient scenario that could be used as an example for when students begin working with patients or communicating with patients who are culturally diverse. Additionally, the nature of the cases created for the course session which were divided into a part A in which the patient physician was more straightforward when diagnosing and treating the patient and a part B with a more comprehensive and nurturing approach to care, allowed the students to compare the scenarios and make their own assumptions and comments on the effectiveness of each portion of the case. Another strength of this training, was the faculty with cultural competence training were uniquely involved in case creation and facilitation of the course session. According to previous studies with similar aims, it was noted that direct observation and feedback from a faculty member who had cultural competence training and direct contact with patients can provide students with a more memorable and useful experience when educating students [12]. The facilitators of this session were able to emphasize from their own personal experiences how to work with culturally diverse populations.

An important aspect of the 2020 iteration of the course session in which a discussion board format was used, was that it allowed students who may feel uncomfortable with sharing their thoughts on a case and their own biases, the opportunity to share in a space that may feel safer than in person [4]. Previous studies have mentioned challenges with online discussion boards [4] but here we had robust participation, albeit required. Students often contributed more than the required number of comments and they were often lengthy and engaging when responding to peers. Finally, in contrast to previous studies, this course session took place in a pre-professional master’s program, the M.S. in Oral Health Sciences Program at Boston University Chobanian & Avedisian School of Medicine. This program, in which students are given the opportunity to enhance their credentials for professional school, provided students with early exposure to cultural competence training. Students that completed this session in their early pre-professional curriculum should be better prepared than peers who did not receive any cultural competence training until they entered their designated professional school. This session is part of an Evidence Based Dentistry course, which incorporates a larger component of personal reflection that serves to engage students in critical thinking as they begin to develop the skills to be future clinicians. Students that understand different cultures, society and themselves through self-assessments will grow and be best suited in time to treat future patients [4, 16, 17].

One limitation of the present study was the number of survey participants that competed the post-session surveys, as survey completion was not required. Thus, the number of student participants declined over the years, reaching its lowest number of participants in 2020 when the discussion board course session was implemented, and students may have been over surveyed due to the pandemic. Another limitation to this study, was the lack of both a pre and post survey that could be used to determine how student’s understanding of cultural competence had evolved from their entry into the course to the conclusion of the course as well as individual bias and self-reporting measures.

In the future, the course should implement both a role-playing format and subsequent discussion board reflections within the same course session. Studies have shown that alternatives ways of drawing students to reflect whether role play, personal narratives, etc. can be extremely advantageous in developing personal reflection and awareness building competency [4, 16–18]. It is noted that role-playing exercises that allow students to provide feedback with student colleagues can provide students with more insight into their own behaviors. It has also been shown in previous studies that student writing and reflection activities can also facilitate student’s reflections on their own beliefs and biases [4, 11]. Reflective writing skills are an important and effective means for students to continue to gauge their cultural competence throughout the remainder of their academic training and as future clinicians [4, 17, 19]. Further, students may experience emotional responses through the process of reflective writing as they recognize personal bias or stereotypes, creating a profound and impactful response resulting in enhanced understanding of cultural differences and beliefs [4]. By combining both learning techniques, students would be able to understand their own bias and their classmates and create a dialogue that could be more beneficial than just one learning method alone. Furthermore, by implementing the discussion board into the role-playing session, as stated previously, students that are more cautious about sharing their point of view or about their own implicit bias in a traditional classroom setting would be able to express their opinions and facilitate a more comprehensive discussion more thoroughly.

## Conclusion

Here we show an effective means to utilize role-play of a multi-scenario case-based patient encounter to teach pre-professional healthcare student’s components of cultural competence, emphasizing the importance of provider-patient interactions, holistic patient care, and patient history and socioeconomic factors in provider care. This study contributes to the larger body of work that seeks to address this important aspect of education as it relates to enhancing patient health care outcomes.

### Electronic supplementary material

Below is the link to the electronic supplementary material.

Supplementary Material 1

Supplementary Material 2

Supplementary Material 3

## Data Availability

The datasets used and/or analyzed during the current study are available from the corresponding author on reasonable request.
