# Supplementary material for: Effectively teaching cultural competence in a pre-professional healthcare curriculum
Source: BMC Med Educ. 2024 May 21;24:553. doi: 10.1186/s12909-024-05507-x (PMC11106880; doi:10.1186/s12909-024-05507-x)
Supplement: Supplementary file 1 — Supplementary Material 1 [file 12909_2024_5507_MOESM1_ESM.docx]

**Additional file 1**

**Title:** Role Play Script – Patient Encounters

**Description:**  Patient encounter cases administered to students as a portion of the case-based, role-playing exercise.

**Patient Encounter Case 1**

**Scenario 1A:**

C: What are your symptoms?

P: I have a lot of pain when I pee.

C: When did it start? How long has this been going on? Have you ever had something like this before?

P: It started 3 days ago and it’s getting worse. This happened the last time I was pregnant too.

C: Do you have any fever or back pain?

P: No.

C: OK, let me look at your urine sample.

C: Well, it looks like you have a UTI. Here’s a prescription for an antibiotic. You should come back for follow-up in 2 weeks.

**Scenario 1B:**

C: What brings you in today?

P: I have a lot of pain when I pee.

C: When did it start? How long has this been going on? Have you ever had something like this before?

P: It started 3 days ago and it’s getting worse. This happened the last time I was pregnant too.

C: How many children do you have?

P: I have a son who is in foster care and then I’m pregnant now with my second kid. Different fathers.

C: Where do you live? Do you live with anyone?

P: I live on the streets because I don’t have a job. I was arrested for drugs a couple of times, and then I lost my son and my job 2 years ago.

C: Do you have any regular care with an obstetrician to follow your pregnancy?

P: Not really. I just go to the ER or come here if I don’t feel good.

C: So, let’s try and make things better for you so you can stay healthy for you and for your baby.

When you pee, it hurts. Do you have any fever or back pain?

P: No.

C: Are you sexually active?

P: Not since I got pregnant.

C: Do you have any vaginal discharge?

P: No.

C: Have you ever been tested for HIV?

P: Yes—about a year ago and it was negative.

C: OK—that’s important information for you and for your baby. Let me look at your urine sample and then we can make a plan together. How does that sound?

P: OK—sounds OK.

C: It looks like you probably have a urinary tract infection. We call it a “UTI”.

P: Yeah—that’s what I had before my son was born.

C: So, you’ll need an antibiotic to treat that infection. We also have to use one that is safe for your unborn baby. We can do that. How do you get your medicines? Do you have insurance?

P: OK—I think I need an antibiotic too. I will have to pay for it because I don’t have insurance. But I don’t have any money right now.

C: OK—so let’s get you samples to complete a full course.

P: Great.

C: I do want to talk to you about other important things regarding your general health.

P: Like what?

C: I would like to refer you to a social worker who could help us figure out a new living situation for you now and for you when the baby arrives.

Being homeless puts you and your baby at risk because it limits your diet and your hygiene.

Could we also arrange for you to see an obstetrician?

P: Sure.

C: I noticed that your teeth are in need of some care. Could we also make an appointment for you to see a dentist?

P: Sure.

C: Great. I would really like to help you. The UTI will be easy to fix. The other things will take some time, but I will help you do that. I want you to come back in 2 weeks to see me, and we can see how the other things are coming along. How does that sound?

P: That sounds good.

**NOTE: The patient does not return for follow-up as planned.**

**Patient Encounter Case 2**

She returns to clinic 3 months after baby is born because of “terrible toothache”.

**Scenario 2A:**

C: What are your symptoms?

P: My tooth is killing me.

C: OK—let’s send you to a dentist.

**Scenario 2B:**

C: What brings you in today?

P: My tooth is killing me.

C: That cannot be fun especially with a new baby. I think we met once when you were pregnant. How are things going?

P: About the same.

C: When did your tooth start to hurt?

P: It probably started after I had the baby, but it is just getting worse and worse. I can’t take the pain at night.

C: What do you use to help with the pain?

P: I am not gonna lie. I am using still and that helps.

C: OK—I am going to be honest here. You need help more than just a dentist to fix this tooth. I am happy to help you work on all of these things, but you have to be willing and have to follow through. Not just for your own health but also for this beautiful baby girl you have now.

P: So, what you do you mean exactly?

C: First, you have a tooth that is infected and may need to be pulled. Your other teeth need attention. So, you need an antibiotic and a dentist to start with.

Most importantly, we need to find you and your baby a safe and clean place to live. This is going to be hard because it means giving up drugs altogether. We can help you all the way along, but you have to be willing to start today.

P: I am. I can’t live like this anymore.
