# Supplementary material for: Effectively teaching cultural competence in a pre-professional healthcare curriculum
Source: BMC Med Educ. 2024 May 21;24:553. doi: 10.1186/s12909-024-05507-x (PMC11106880; doi:10.1186/s12909-024-05507-x)
Supplement: Supplementary file 2 — Supplementary Material 2 [file 12909_2024_5507_MOESM2_ESM.docx]

**Additional file 3**

**Format:** MS Word (.docx)

**Title:** Cultural Competence Sample Survey

**Description:**  Post-session survey administered at the conclusion of the cultural competence training sessions.

Sample Survey

Q1 Please indicate your level of agreement with each statement below regarding the Communication & Cultural Competence Role Play Case.

|  | Strongly Disagree | Disagree | Agree | Strongly Agree |
| --- | --- | --- | --- | --- |
| The Communication & Cultural Competence Role Play exercise was enjoyable. |  |  |  |  |
| The role play exercises were helpful in identifying the importance of communication in patient encounters. |  |  |  |  |
| The role play exercises made me more aware of different strategies used to improve patient interview skills. |  |  |  |  |
| I am better able to identify the importance of building rapport and trust during patient contact. |  |  |  |  |
| The role play exercises helped me to understand my own bias and/or cultural awareness when working with patients. |  |  |  |  |

Q2 Please add any additional comments on the Communication & Cultural Competence Case

________________________________________________________________

________________________________________________________________

________________________________________________________________

________________________________________________________________

Q3 Please indicate your level of agreement with each statement below regarding the Communication & Cultural Competence discussion board case.

|  | Strongly Disagree | Disagree | Agree | Strongly Agree |
| --- | --- | --- | --- | --- |
| The Communication & Cultural Competence discussion board exercise was enjoyable. |  |  |  |  |
| The discussion board exercises were helpful in identifying the importance of communication in patient encounters. |  |  |  |  |
| The discussion board exercises helped me to understand different strategies to improve patient interview skills. |  |  |  |  |
| I am better able to identify the importance of building rapport and trust during patient contact. |  |  |  |  |
| The discussion board exercises helped me to understand my own bias and/or cultural awareness when working with patients. |  |  |  |  |

Q4 Please add any additional comments on the Communication & Cultural Competence Case and your experience using the discussion Blackboard discussion board.

________________________________________________________________

________________________________________________________________

________________________________________________________________

________________________________________________________________
