# Supplementary material for: Effectively teaching cultural competence in a pre-professional healthcare curriculum
Source: BMC Med Educ. 2024 May 21;24:553. doi: 10.1186/s12909-024-05507-x (PMC11106880; doi:10.1186/s12909-024-05507-x)
Supplement: Supplementary file 3 — Supplementary Material 3 [file 12909_2024_5507_MOESM3_ESM.pptx]

## Slide 1
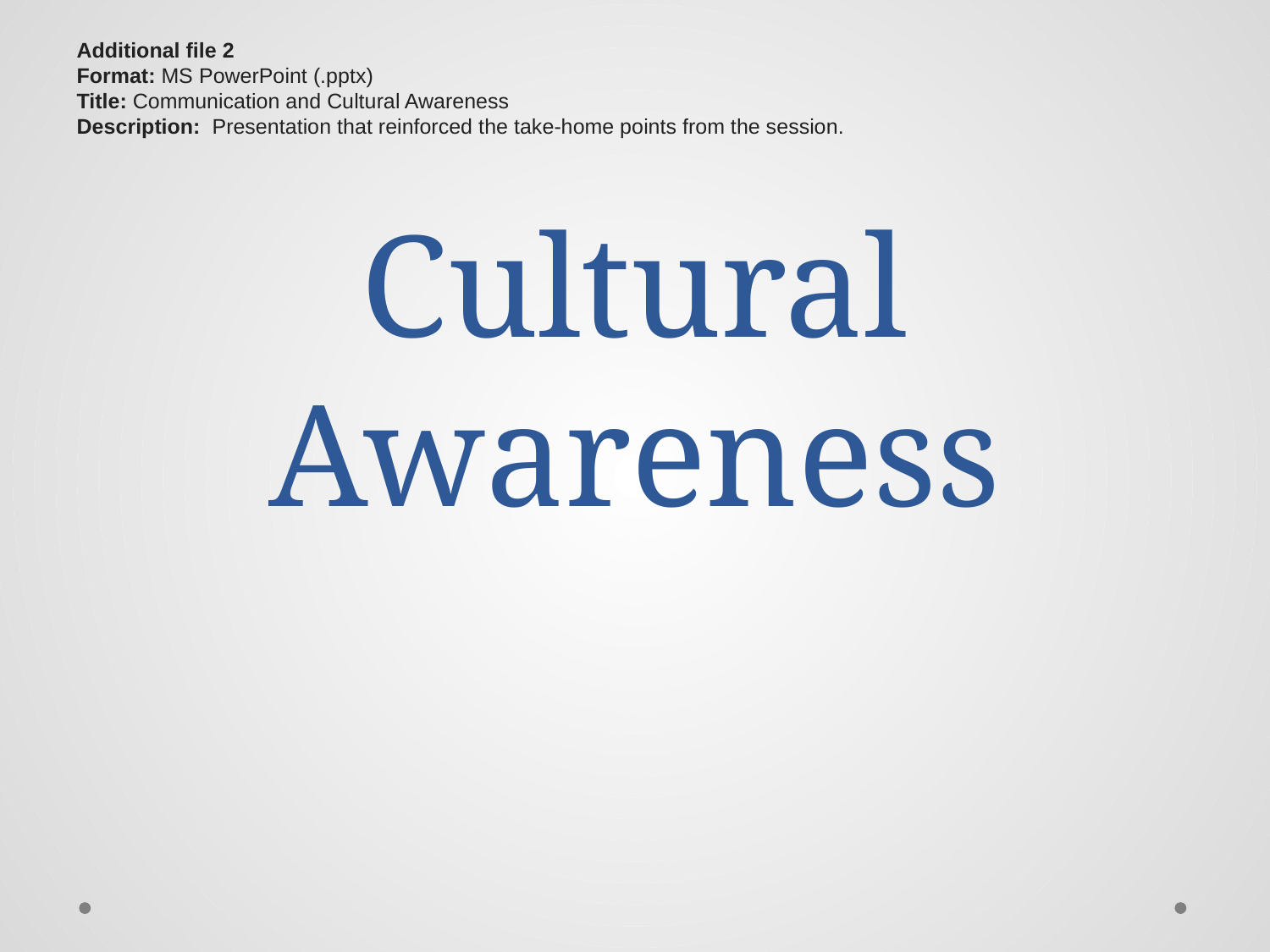

Additional file 2
Format: MS PowerPoint (.pptx)
Title: Communication and Cultural Awareness
Description:  Presentation that reinforced the take-home points from the session.
# Cultural Awareness

## Slide 2
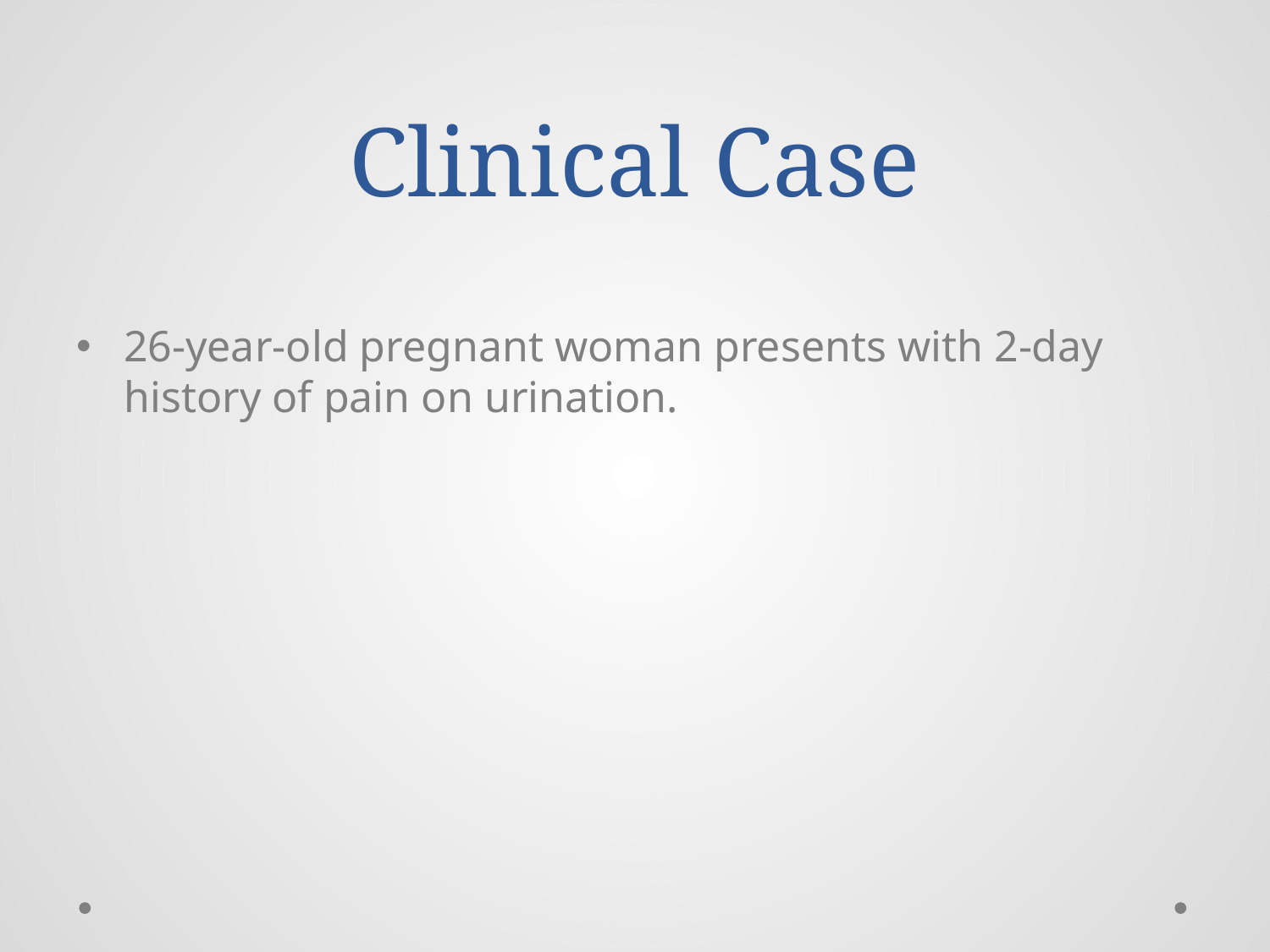

# Clinical Case
26-year-old pregnant woman presents with 2-day history of pain on urination.

## Slide 3
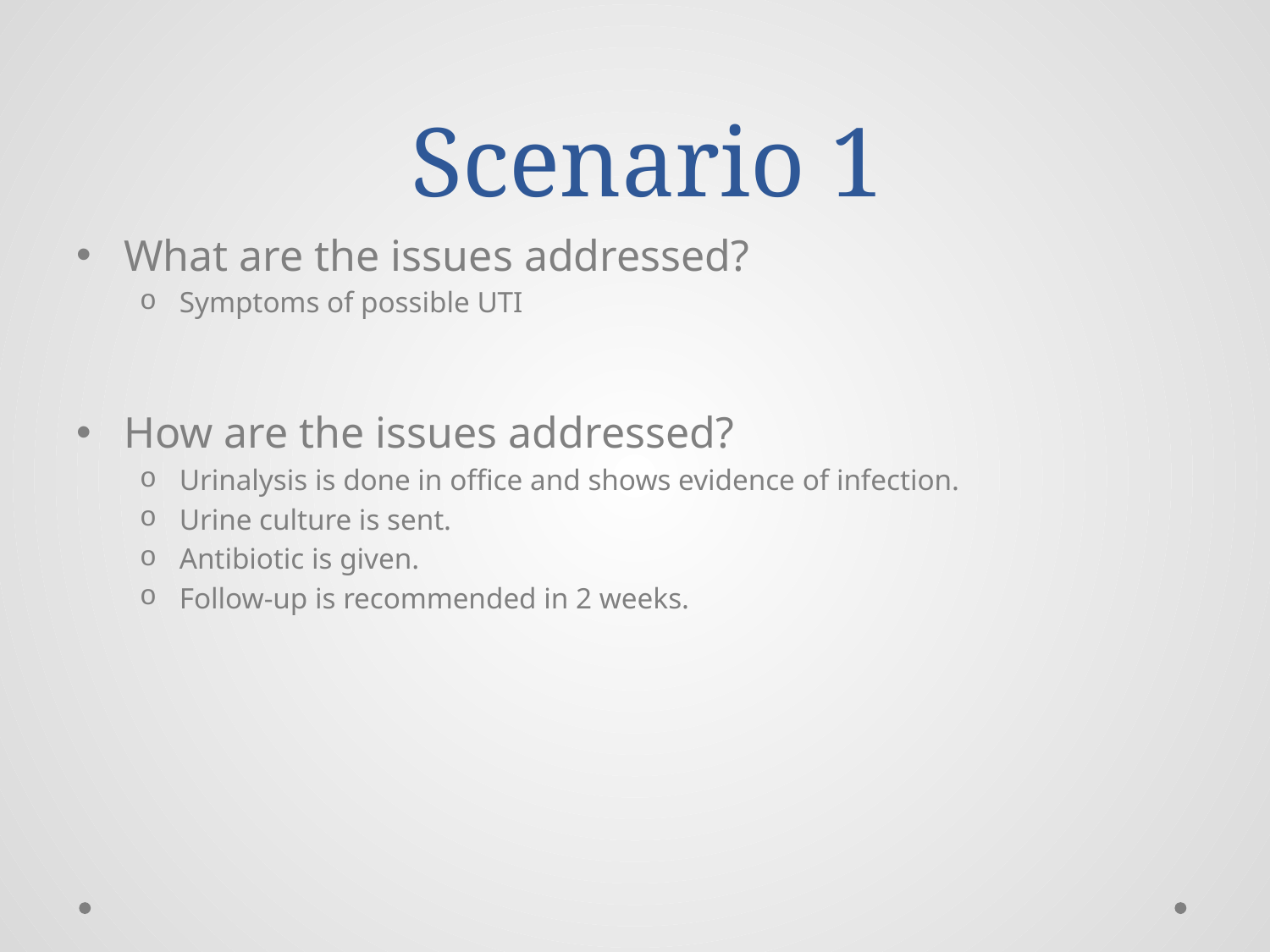

# Scenario 1
What are the issues addressed?
Symptoms of possible UTI
How are the issues addressed?
Urinalysis is done in office and shows evidence of infection.
Urine culture is sent.
Antibiotic is given.
Follow-up is recommended in 2 weeks.

## Slide 4
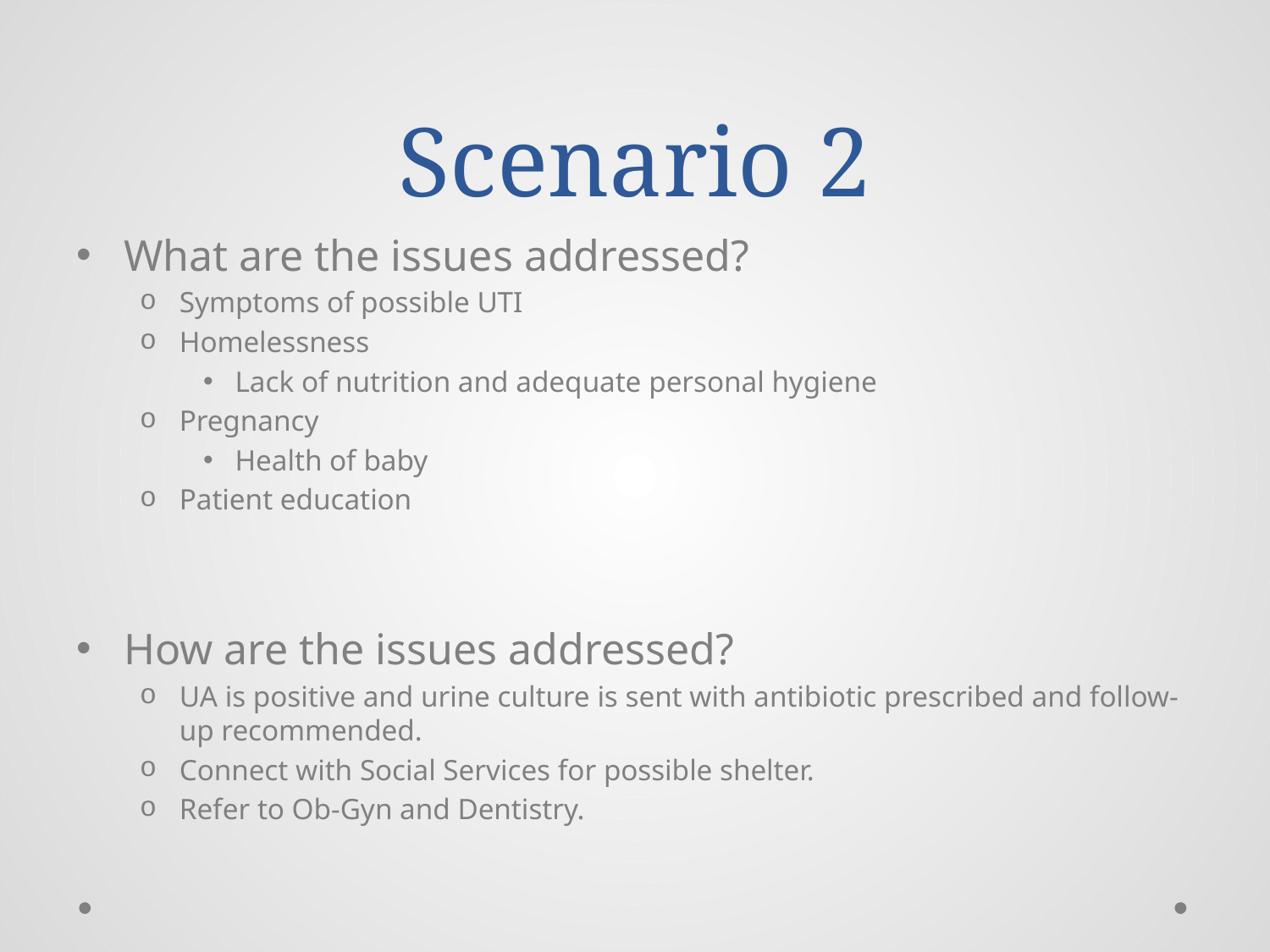

# Scenario 2
What are the issues addressed?
Symptoms of possible UTI
Homelessness
Lack of nutrition and adequate personal hygiene
Pregnancy
Health of baby
Patient education
How are the issues addressed?
UA is positive and urine culture is sent with antibiotic prescribed and follow-up recommended.
Connect with Social Services for possible shelter.
Refer to Ob-Gyn and Dentistry.

## Slide 5
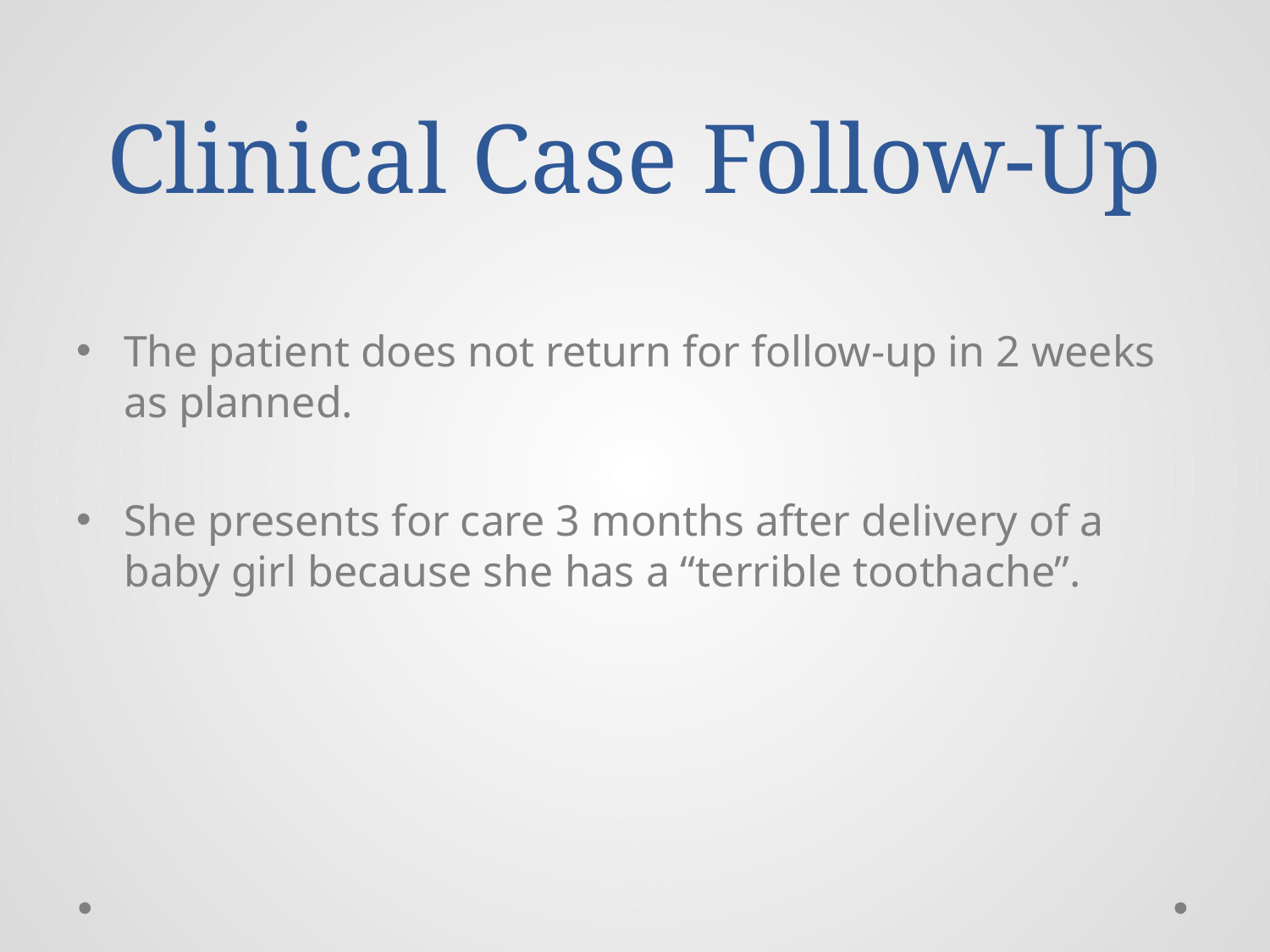

# Clinical Case Follow-Up
The patient does not return for follow-up in 2 weeks as planned.
She presents for care 3 months after delivery of a baby girl because she has a “terrible toothache”.

## Slide 6
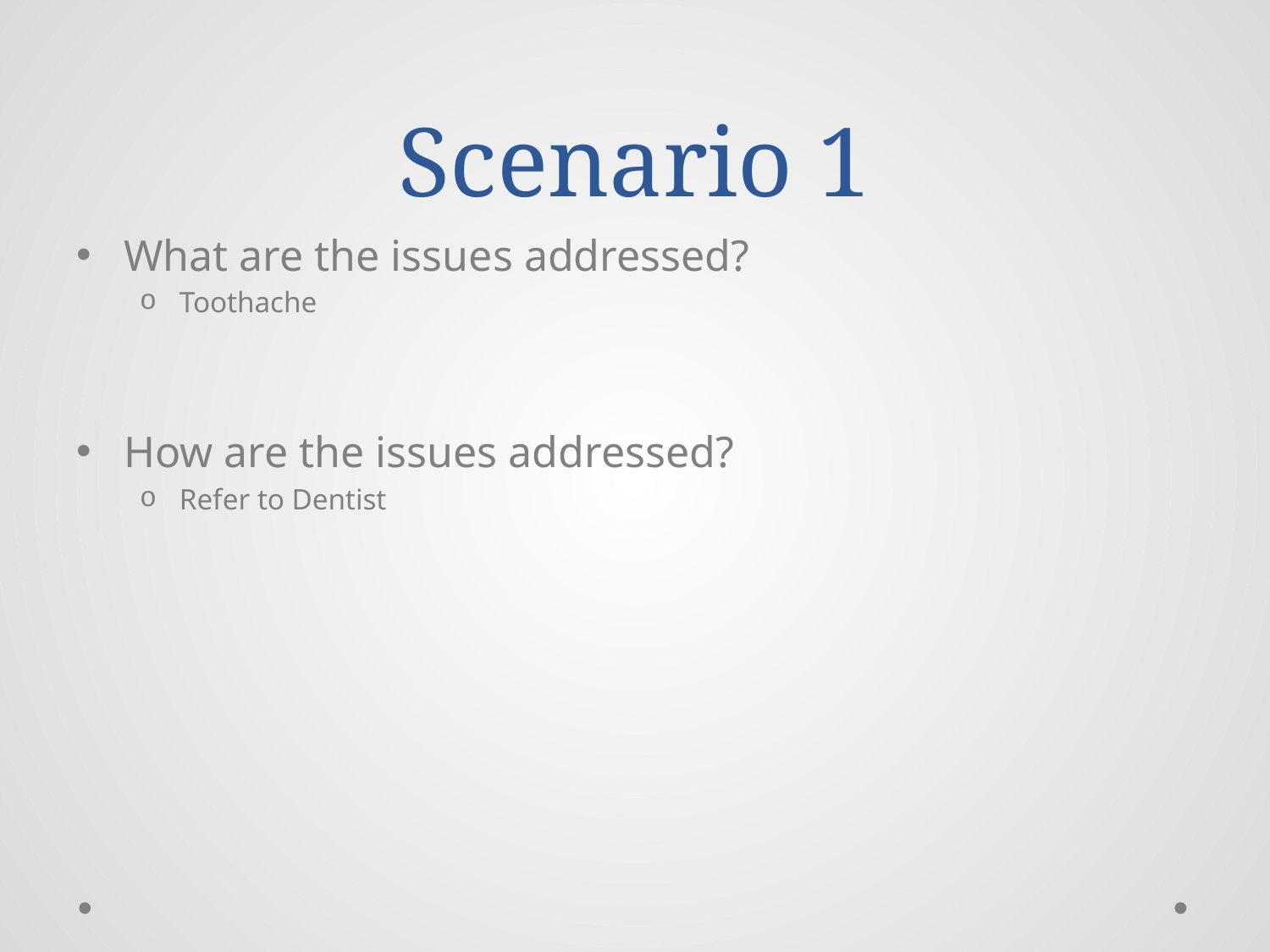

# Scenario 1
What are the issues addressed?
Toothache
How are the issues addressed?
Refer to Dentist

## Slide 7
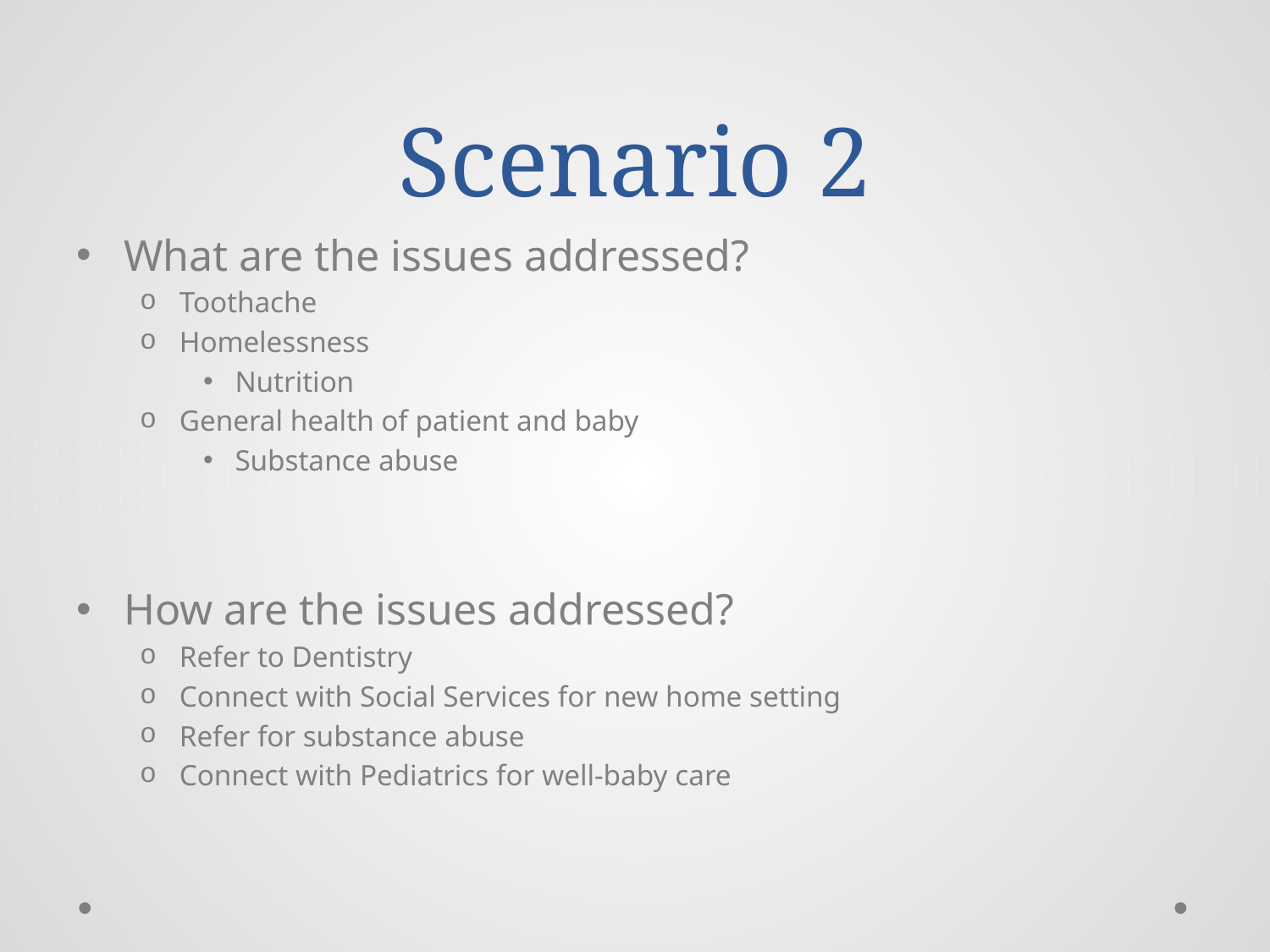

# Scenario 2
What are the issues addressed?
Toothache
Homelessness
Nutrition
General health of patient and baby
Substance abuse
How are the issues addressed?
Refer to Dentistry
Connect with Social Services for new home setting
Refer for substance abuse
Connect with Pediatrics for well-baby care

## Slide 8
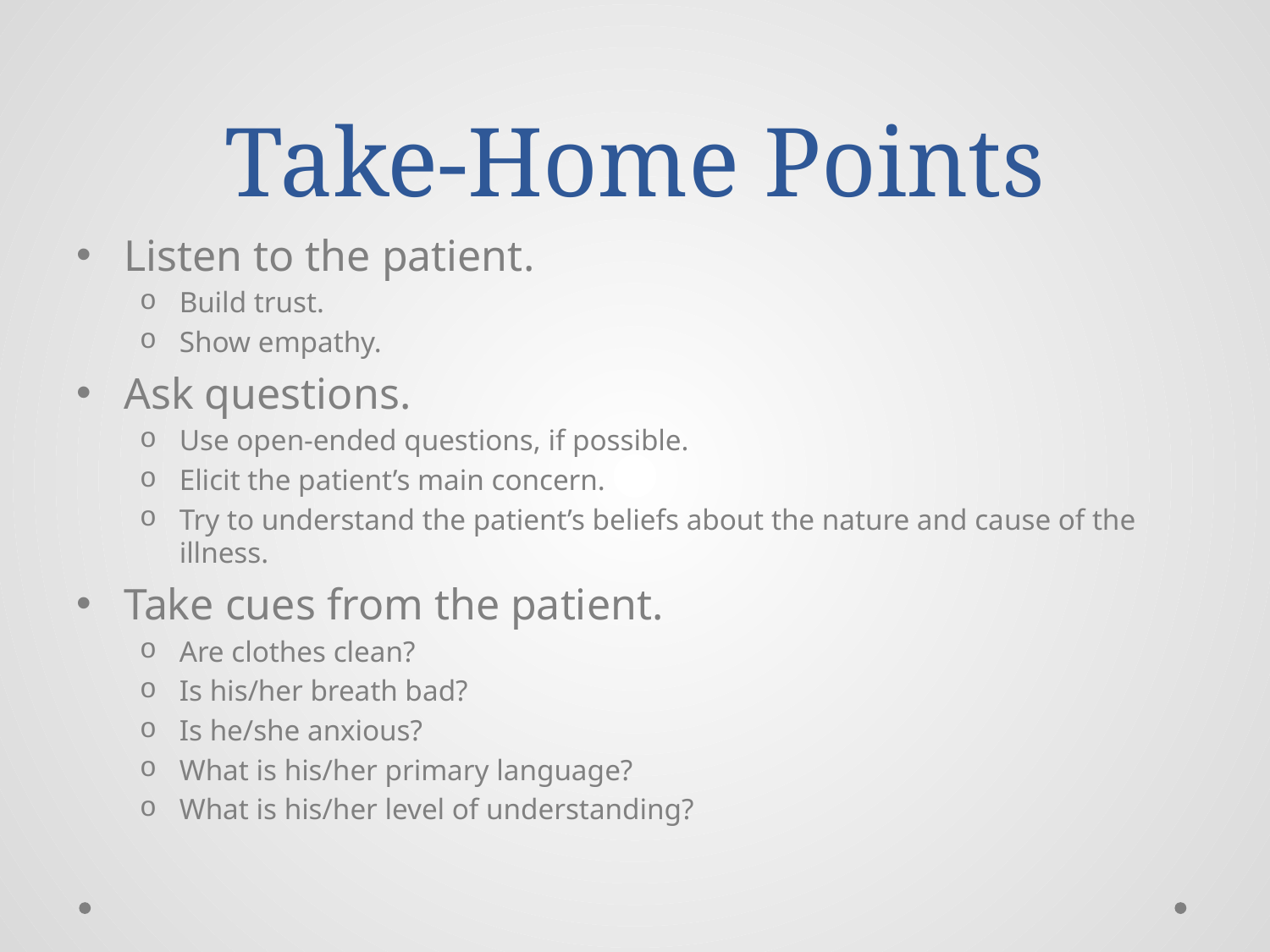

# Take-Home Points
Listen to the patient.
Build trust.
Show empathy.
Ask questions.
Use open-ended questions, if possible.
Elicit the patient’s main concern.
Try to understand the patient’s beliefs about the nature and cause of the illness.
Take cues from the patient.
Are clothes clean?
Is his/her breath bad?
Is he/she anxious?
What is his/her primary language?
What is his/her level of understanding?

## Slide 9
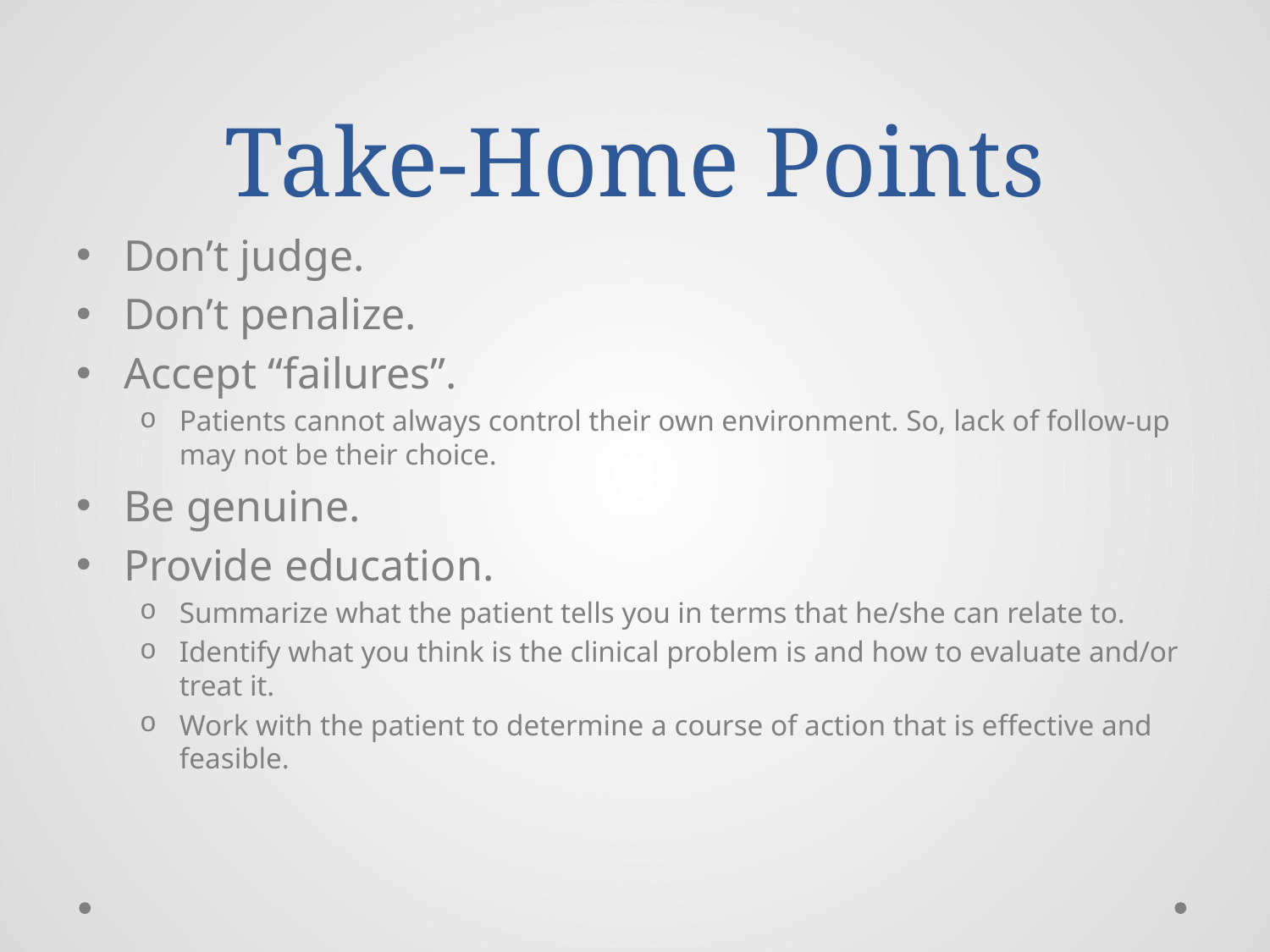

# Take-Home Points
Don’t judge.
Don’t penalize.
Accept “failures”.
Patients cannot always control their own environment. So, lack of follow-up may not be their choice.
Be genuine.
Provide education.
Summarize what the patient tells you in terms that he/she can relate to.
Identify what you think is the clinical problem is and how to evaluate and/or treat it.
Work with the patient to determine a course of action that is effective and feasible.

## Slide 10
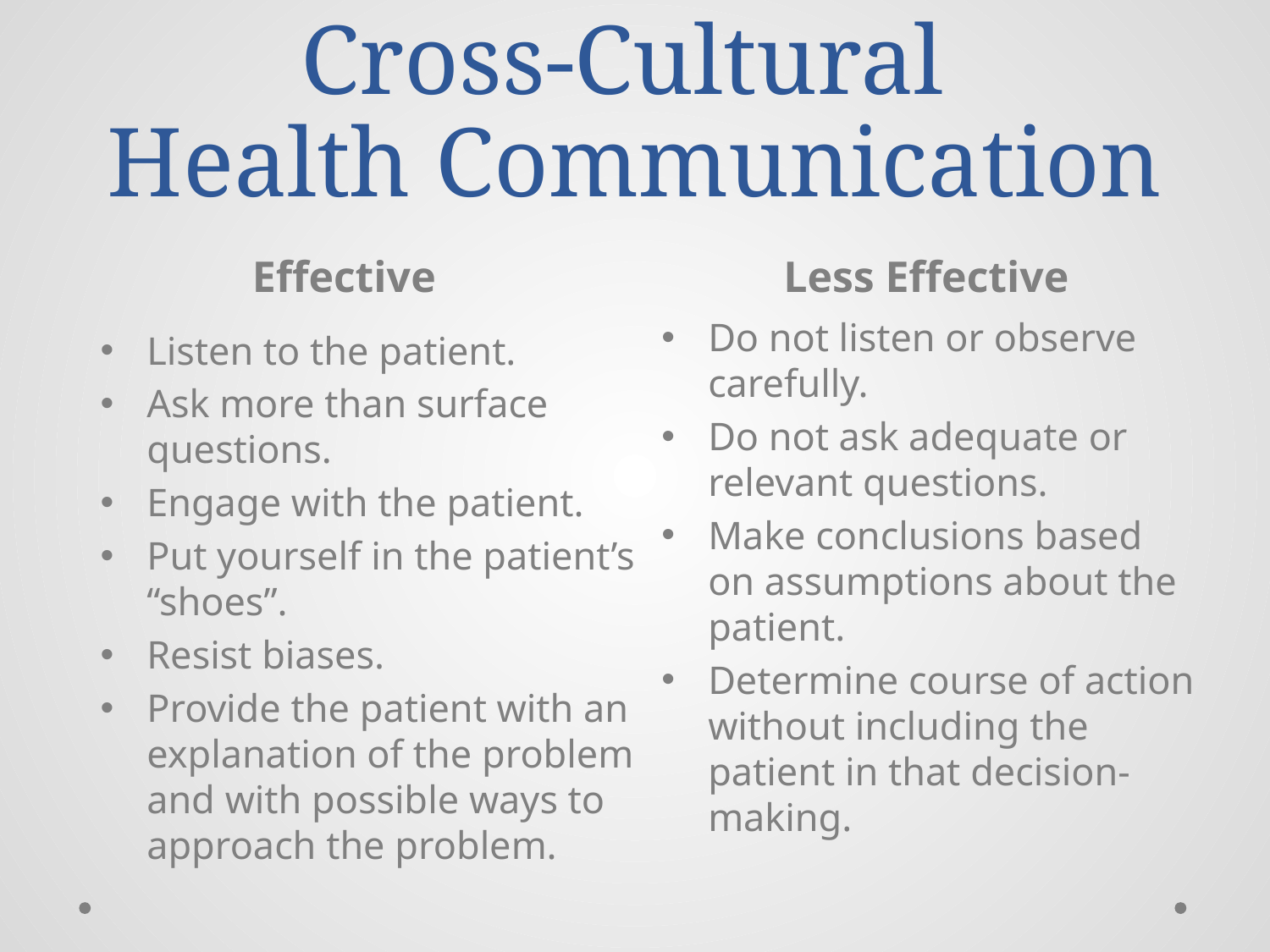

# Cross-Cultural Health Communication
Effective
Less Effective
Do not listen or observe carefully.
Do not ask adequate or relevant questions.
Make conclusions based on assumptions about the patient.
Determine course of action without including the patient in that decision-making.
Listen to the patient.
Ask more than surface questions.
Engage with the patient.
Put yourself in the patient’s “shoes”.
Resist biases.
Provide the patient with an explanation of the problem and with possible ways to approach the problem.

## Slide 11
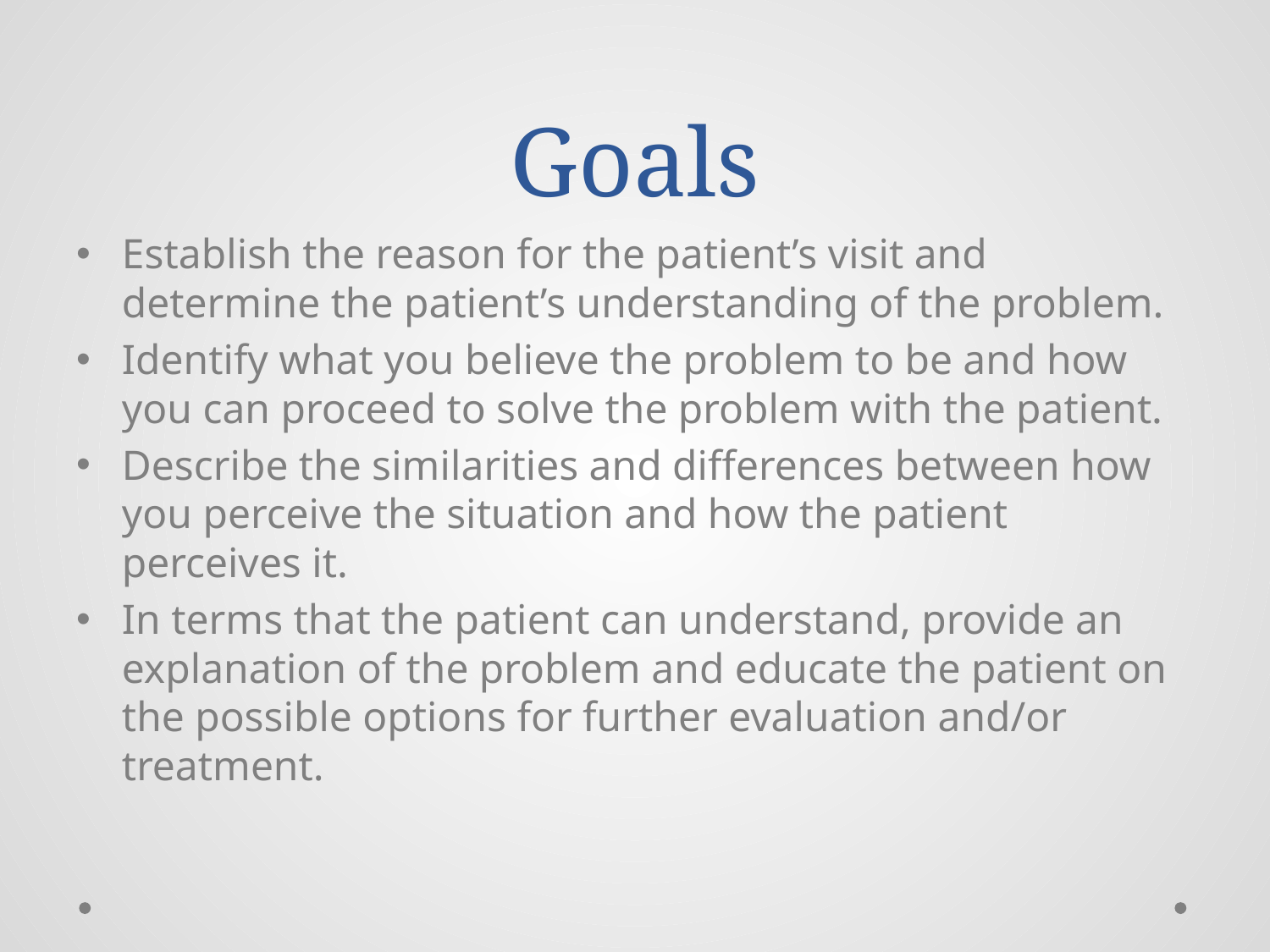

# Goals
Establish the reason for the patient’s visit and determine the patient’s understanding of the problem.
Identify what you believe the problem to be and how you can proceed to solve the problem with the patient.
Describe the similarities and differences between how you perceive the situation and how the patient perceives it.
In terms that the patient can understand, provide an explanation of the problem and educate the patient on the possible options for further evaluation and/or treatment.
